# Supplementary material for: Quality of Life (QoL) in Patients with Chronic Inflammatory Bowel Diseases: How Much Better with Biological Drugs?
Source: J Pers Med. 2023 Jun 2;13(6):947. doi: 10.3390/jpm13060947 (PMC10302043; doi:10.3390/jpm13060947)
Supplement: Supplementary file 1 [file jpm-13-00947-s001.zip › jpm-2309068-supplementary.pdf]

Supplementary figures

Figure S1. Box and whisker plots representing the SF-36 main domains, and the baseline, T1, and T2 are compared. *p* < 0.001 for all comparisons. Comparisons were carried out using the Friedman test.

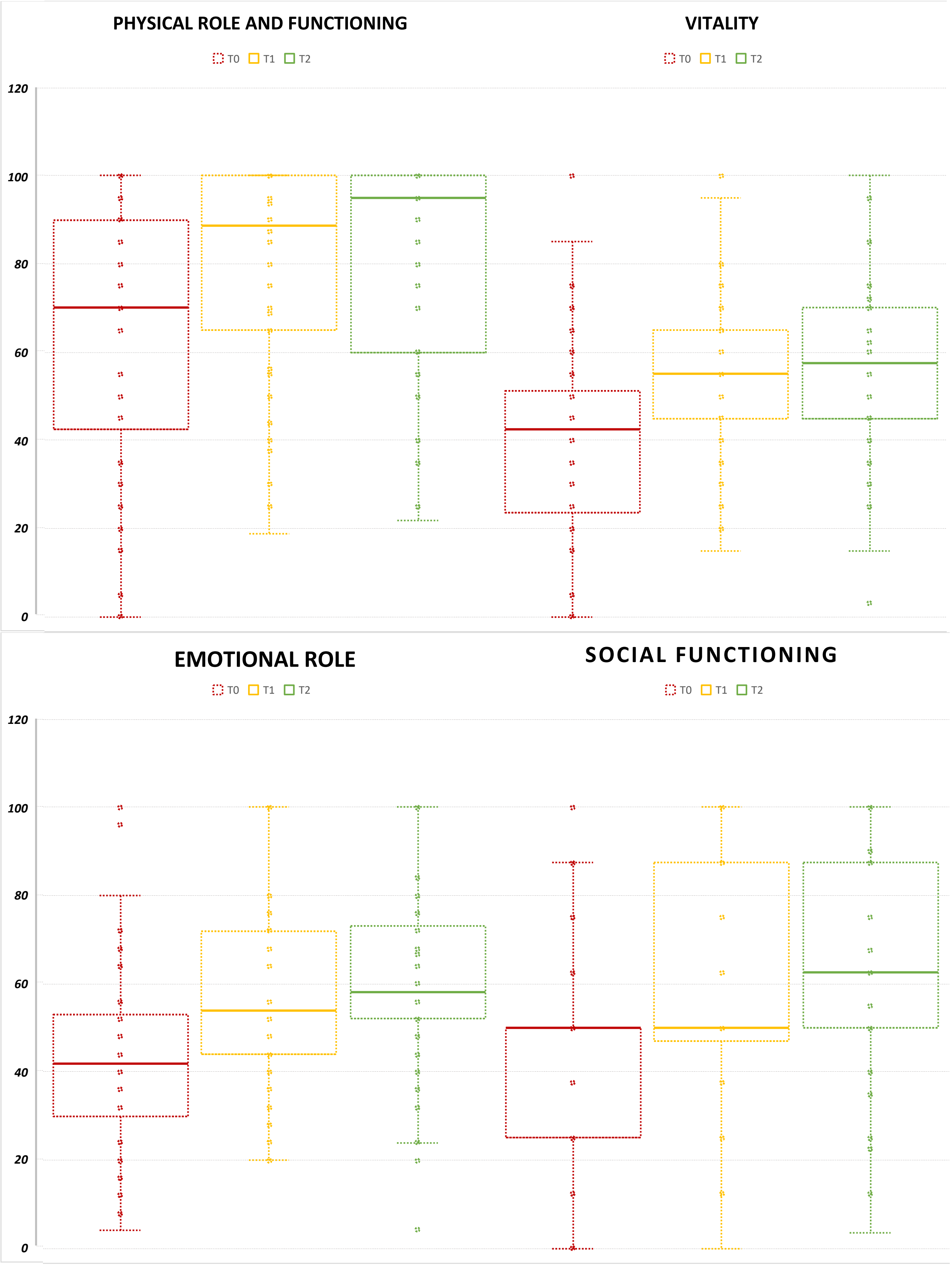

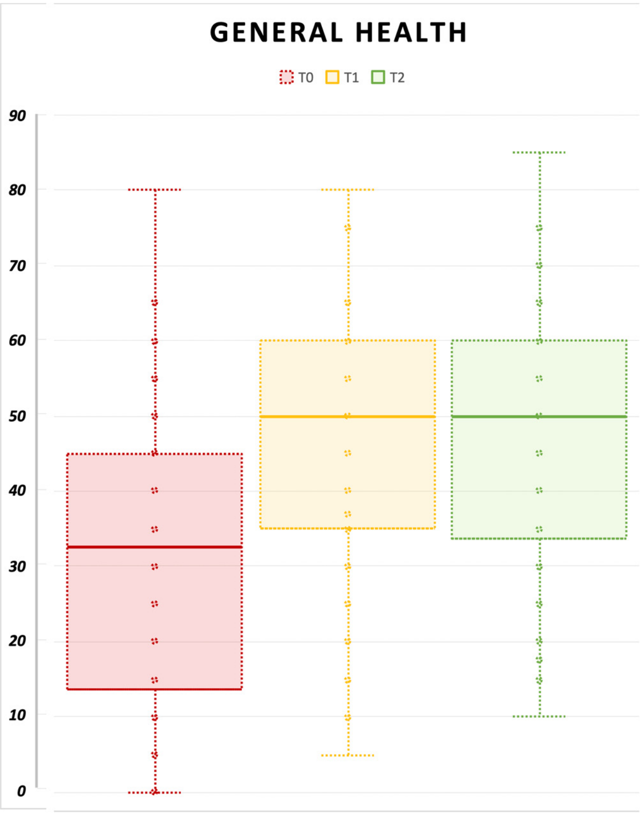

Figure S2. Box and whisker plots representing the FACIT main domains. The baseline, T1, and T2 are compared. $p < 0.001$ for all comparisons. Comparisons were carried out using the Friedman test.

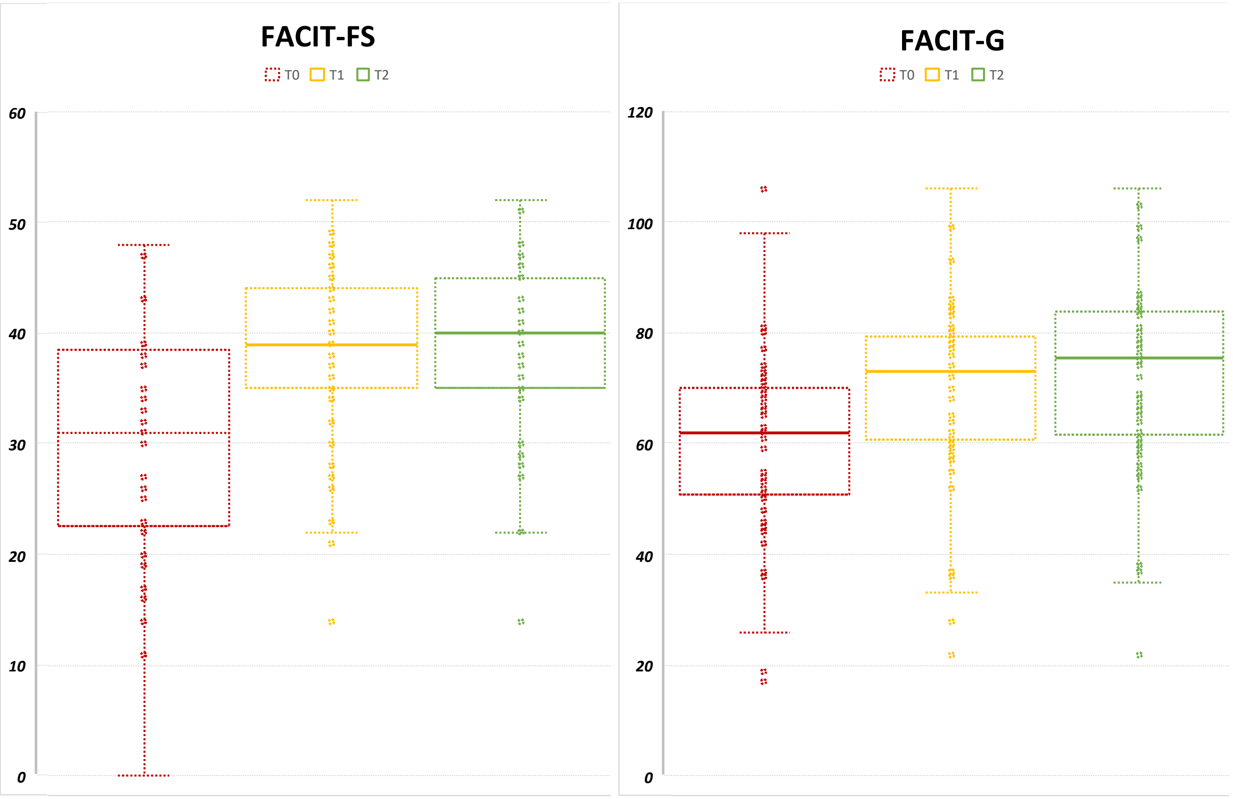

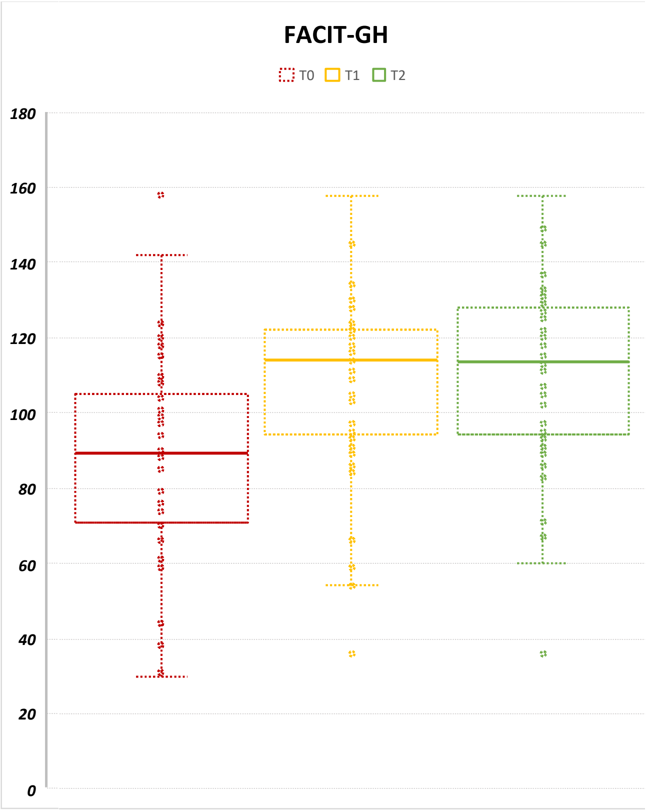

Figure S3. Box and whisker plots representing the WPAI:HG main domains. Upper panel: work activity impairment score; lower panel: regular activities impairment score comparing the baseline, T1, and T2. $p < 0.01$ and $p < 0.001$, respectively; comparisons were carried out using the Friedman test.

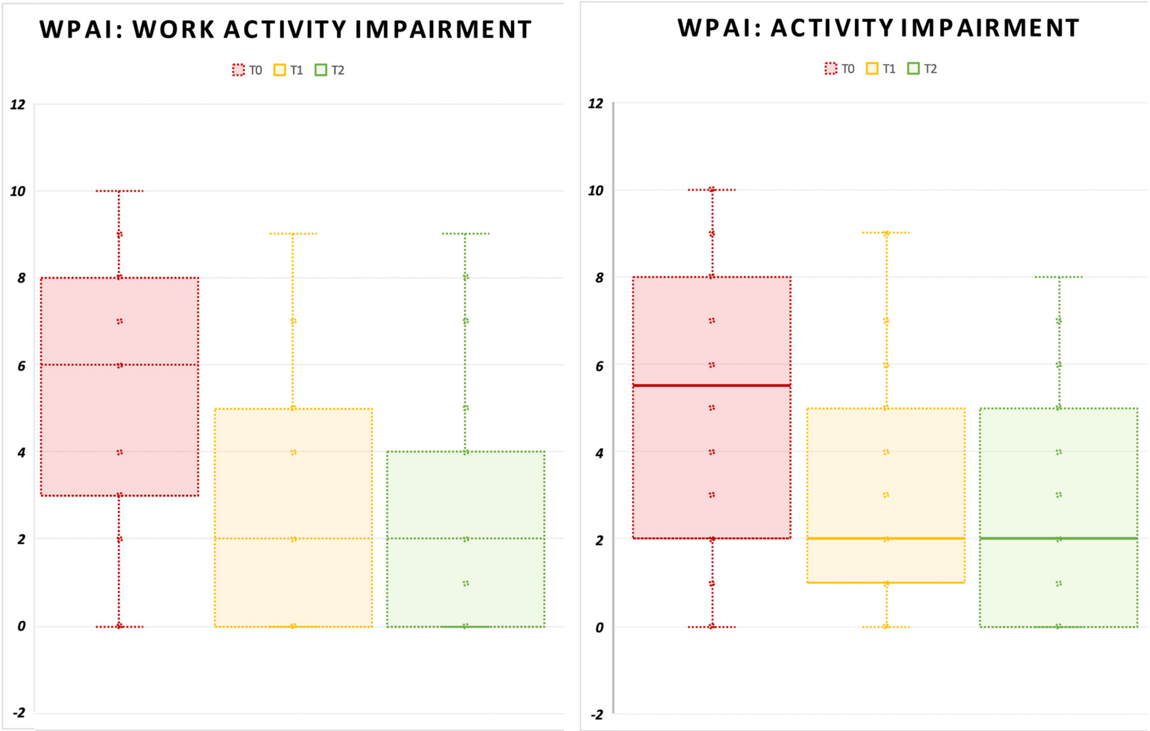

Table S1: Spearman’s interrelationships between delta change percentages of HRQoL questionnaire items each other.

		SF36_1	SF36_2	SF36_3	SF36_4	SF36_5	SF36_6	SF36_7	SF36_8	FACIT_1_	FACIT_2	FACIT_3	FACIT_4	FACIT_5	FACIT_6	FACIT_7	WPAI_2	WPAI_3	WPAI_4	WPAI_5	WPAI_6
**SF36_1**	ρ	1.000	0.335	0.387	0.523	0.425	0.312	0.507	0.516	0.704	0.091	0.354	0.474	0.604	0.724	0.775	-0.665	0.192	0.322	-0.253	-0.502
	p		<0.01	0.007	<0.001	0.003	<0.05	<0.001	<0.001	<0.001	ns	<0.01	<0.001	<0.001	<0.001	<0.001	<0.001	ns	<0.100	ns	<0.01
**SF36_2**	ρ		1.000	0.383	0.549	0.423	0.565	0.300	0.515	0.335	-0.355	0.096	0.125	0.435	0.055	0.183		-0.211	-0.424	-0.912	-0.567
	p			<0.005	<0.001	<0.005	<0.001	<0.05	<0.001	<0.01	<0.01	ns	ns	<0.005	ns	ns		ns	<0.025	<0.001	<0.005
**SF36_3**	ρ			1.000	0.505	0.468	0.604	0.417	0.375	0.318	0.007	0.292	0.294	0.352	0.323	0.415	0.087	0.226	0.221	-0.174	-0.361
	p				<0.001	<0.001	<0.001	<0.005	<0.005	<0.05	ns	<0.05	<0.05	<0.01	<0.05	<0.005	ns	ns	ns	ns	<0.05
**SF36_4**	ρ				1.000	0.672	0.566	0.667	0.442	0.530	-0.297	0.298	0.303	0.565	0.343	0.502	-0.170	0.410	0.218	-0.203	-0.528
	p					<0.001	<0.001	<0.001	<0.001	<0.001	<0.05	<0.05	<0.05	<0.001	<0.01	<0.001	ns	<0.05	ns	ns	<0.01
**SF36_5**	ρ					1.000	0.554	0.515	0.561	0.401	-0.256	0.332	0.240	0.392	0.337	0.461	-0.503	-0.214	-0.102	-0.477	-0.361
	p						<0.001	<0.001	<0.001	<0.001	<0.05	<0.01	<0.05	<0.005	<0.01	<0.001	<0.01	ns	ns	<0.025	0.05
**SF36_6**	ρ						1.000	0.500	0.452	0.213	-0.176	0.392	0.301	0.353	0.273	0.373	-0.289	-0.374	-0.280	-0.435	-0.439
	p							<0.001	<0.001	ns	ns	<0.005	<0.05	<0.01	<0.05	<0.005	<0.10	<0.05	<0.100	<0.025	<0.025
**SF36_7**	ρ							1.000	0.335	0.607	-0.094	0.098	0.243	0.358	0.476	0.502	-0.643	0.100	0.018	-0.336	-0.499
	p								<0.01	<0.001	ns	ns	<0.05	<0.01	<0.001	<0.001	<0.001	ns	ns	<0.05	<0.01
**SF36_8**	ρ								1.000	0.289	-0.170	0.343	0.292	0.402	0.318	0.425	-0.701	-0.035	-0.142	-0.331	-0.269
	p									<0.05	ns	<0.01	<0.05	<0.005	<0.05	<0.005	<0.001	ns	ns	<0.100	Ns
**FACIT_1**	ρ									1.000	0.081	0.386	0.432	0.518	0.834	0.778	-0.037	-0.039	0.243	-0.269	-0.535
	p										ns	<0.005	<0.005	<0.001	<0.001	<0.001	ns	ns	ns	ns	<0.005
**FACIT_2**	ρ										1.000	0.056	-0.046	0.137	0.270	0.238	0.153	0.356	0.503	0.168	0.028
	p											ns	ns	Ns	<0.05	<0.05	ns	<0.05	<0.01	ns	Ns
**FACIT_3**	ρ											1.000	0.461	0.535	0.558	0.597	-0.026	-0.397	-0.296	-0.349	-0.083
	p												<0.001	<0.001	<0.001	<0.001	ns	<0.05	<0.100	<0.100	Ns
**FACIT_4**	ρ												1.000	0.419	0.572	0.532	0.168	-0.200	0.181	0.016	-0.181
	p													<0.005	<0.001	<0.001	ns	ns	ns	ns	Ns
**FACIT_5**	ρ													1.000	0.538	0.803	-0.548	-0.037	0.071	-0.533	-0.419
	p														<0.001	<0.001	<0.005	ns	ns	<0.005	<0.025
**FACIT_6**	ρ														1.000	0.915	-0.075	-0.167	0.158	-0.259	-0.479
	p															<0.001	ns	ns	ns	ns	<0.025
**FACIT_7**	ρ															1.000	-0.410	-0.225	0.104	-0.439	-0.492
	p																<0.05	ns	ns	<0.025	<0.01
**WPAI_2**	ρ																1.000	0.194	0.459	0.603	0.694
	p																	ns	<0.025	<0.005	<0.001
**WPAI_3**	ρ																	1.000	0.938	0.305	-0.060
	p																		<0.001	<0.100	Ns
**WPAI_4**	ρ																		1.000	0.222	-0.157
	p																			ns	Ns
**WPAI_5**	ρ																			1.000	0.715
	p																				<0.001
**WPAI_6**	ρ																				1.000
		**SF36_1**	**SF36_2**	**SF36_3**	**SF36_4**	**SF36_5**	**SF36_6**	**SF36_7**	**SF36_8**	**FACIT_1**	**FACIT_2**	**FACIT_3**	**FACIT_4**	**FACIT_5**	**FACIT_6**	**FACIT_7**	**WPAI_1**	**WPAI_2**	**WPAI_3**	**WPAI_4**	**WPAI_5**

SF-36 items: SF36_1, Physical functioning; SF36_2, Role limitations due to physical health; SF36_3, Role limitations due to emotional problems; SF36_4, Energy/fatigue; SF36_5, Emotional wellbeing; SF36_6, Social Functioning; SF36_7, Pain; SF36_8, General health.
FACIT items: FACIT_1, Physical wellbeing; FACIT_2, Social/family wellbeing; FACIT_3, Emotional wellbeing; FACIT_4, Functional wellbeing; FACIT_5, Additional Concerns; FACIT_6, FACIT-G), and the, obtained summing the FACIT-G with the FACIT-FS; FACIT_7, global FACIT score (FACIT-GH).
WPAI items: WPAI_2, work hours missed because of health problems; WPAI_3, work hours missed because of any other reason; WPAI_4, last seven days worked hours; WPAI_5, last seven days impairment in work activities; WPAI_6, last seven days impairment in regular activities.

Table S2: Spearman's interrelationships between delta change percentages of main serum markers and HRQoL questionnaire items.

		**WBC**	**N**	**L**	**Alpha1**	**Alpha2**	**ESR**	**CRP**	**Hb**	**MCV**	**MCH**	**RBC**
**SF36_1**	ρ	-0.097	0.165	-0.263	0.028	0.034	0.114	0.301	-0.075	-0.170	-0.137	0.086
	p	Ns	ns	<0.05	ns	ns	ns	<0.005	ns	ns	ns	ns
**SF36_2**	ρ	0.418	0.555	-0.496	0.038	0.141	0.232	0.580	0.081	-0.167	-0.111	0.257
	p	<0.05	<0.001	<0.001	ns	ns	ns	<0.001	ns	ns	ns	<0.05
**SF36_3**	ρ	-0.174	-0.247	0.207	-0.158	-0.002	-0.167	-0.009	-0.116	-0.112	0.037	-0.085
	p	Ns	<0.05	ns	ns	ns	ns	ns	ns	ns	ns	ns
**SF36_4**	ρ	0.006	-0.015	-0.005	-0.106	-0.084	0.076	0.072	0.020	-0.095	-0.077	0.036
	p	Ns	ns	ns	ns	ns	ns	ns	ns	ns	ns	ns
**SF36_5**	ρ	0.137	0.091	-0.151	-0.241	0.014	-0.040	0.315	-0.311	-0.248	-0.349	-0.060
	p	Ns	ns	ns	<0.05	ns	ns	<0.05	<0.01	<0.05	<0.01	ns
**SF36_6**	ρ	0.145	-0.175	0.218	-0.261	-0.116	-0.036	-0.034	-0.127	-0.025	-0.157	0.071
	p	Ns	ns	ns	<0.05	ns	ns	ns	ns	ns	Ns	ns
**SF36_7**	ρ	0.124	0.057	-0.082	0.028	0.173	0.160	0.098	-0.129	-0.151	-0.175	-0.014
	p	Ns	ns	ns	ns	ns	ns	ns	ns	ns	Ns	ns
**SF36_8**	ρ	0.108	0.194	-0.223	-0.136	-0.011	-0.031	0.230	-0.177	-0.254	-0.266	0.068
	p	Ns	ns	ns	ns	ns	ns	ns	ns	<0.05	<0.05	ns
**FACIT_1**	ρ	-0.029	0.169	-0.232	-0.029	0.129	0.013	0.370	0.032	-0.348	-0.219	0.173
	p	Ns	ns	ns	ns	ns	ns	<0.005	ns	<0.01	Ns	ns
**FACIT_2**	ρ	-0.213	-0.171	0.129	0.118	0.125	0.037	0.116	-0.061	-0.045	0.090	-0.110
	p	Ns	ns	ns	ns	ns	ns	Ns	ns	ns	Ns	ns
**FACIT_3**	ρ	-0.008	-0.163	0.128	-0.193	-0.012	0.008	0.236	0.011	-0.178	-0.118	0.045
	p	Ns	ns	ns	ns	ns	ns	=0.05	ns	ns	Ns	ns
**FACIT_4**	ρ	0.109	-0.061	-0.009	-0.043	0.011	-0.227	0.055	0.149	-0.209	-0.157	0.233
	p	Ns	ns	ns	ns	ns	ns	Ns	ns	ns	ns	ns
**FACIT_5**	ρ	-0.093	0.069	-0.092	0.022	-0.062	0.024	0.123	-0.040	-0.231	-0.143	0.046
	p	Ns	ns	ns	ns	ns	ns	Ns	ns	ns	ns	ns
**FACIT_6**	ρ	-0.021	0.060	-0.116	-0.037	0.165	-0.018	0.303	0.003	-0.344	-0.196	0.087
	p	Ns	ns	ns	ns	ns	ns	<0.01	ns	<0.01	ns	ns
**FACIT_7**	ρ	-0.096	0.056	-0.124	-0.065	0.079	-0.005	0.229	-0.032	-0.369	-0.220	0.074
	p	Ns	ns	ns	ns	ns	ns	Ns	ns	<0.005	ns	ns
**WPAI_2**	ρ	-0.075	-0.596	0.601	-0.056	-0.242	-0.335	-0.242	0.617	0.634	0.857	0.019
	p	Ns	<0.005	<0.005	ns	ns	<0.100	Ns	<0.005	<0.001	<0.001	ns
**WPAI_3**	ρ	-0.515	-0.252	0.204	0.488	0.143	0.157	0.248	0.205	0.140	0.249	0.092
	p	<0.01	ns	ns	<0.01	ns	ns	Ns	ns	ns	ns	ns
**WPAI_4**	ρ	-0.437	-0.149	0.055	0.375	0.053	-0.089	0.198	0.169	-0.048	0.070	0.113

	p	<0.025	ns	ns	<0.05	ns	ns	ns	ns	ns	ns	ns
**WPAI_5**	ρ	-0.496	-0.323	0.208	0.317	-0.244	-0.049	-0.130	-0.040	0.260	0.361	-0.162
	p	<0.01	<0.100	ns	<0.100	ns	ns	ns	ns	ns	<0.05	ns
**WPAI_6**	ρ	-0.198	-0.253	0.199	0.008	-0.150	-0.162	-0.230	-0.045	0.248	0.203	-0.159
	p	Ns	ns	ns	ns	ns	ns	ns	ns	ns	ns	ns
		**WBC**	**N**	**L**	**Alpha1**	**Alpha2**	**ESR**	**CRP**	**Hb**	**MCV**	**MCH**	**RBC**

Abbreviations: ρ: correlation coefficient; WBC: white blood cells; N: neutrophils; L: lymphocytes; Alpha1: alpha1 globulins; Alpha2: alpha2 globulins; ESR: erythrocytes sedimentation rate; CRP: C-reactive protein; Hb: hemoglobin; MCV: mean corpuscular volume; MCH: mean corpuscular hemoglobin; RBC: red blood cells.
